# Gold Standard Program for Heavy Smokers in a Real-Life Setting

**DOI:** 10.3390/ijerph10094186

**Published:** 2013-09-09

**Authors:** Tim Neumann, Mette Rasmussen, Berit L. Heitmann, Hanne Tønnesen

**Affiliations:** 1 Clinical Health Promotion Centre, Department of Health Sciences, Lund University, Skåne University Hospital, Entrance. 108, Malmö SE 205 02, Sweden; E-Mail: hanne.tonnesen@med.lu.se; 2 WHO-CC Clinical Health Promotion Centre, Bispebjerg and Frederiksberg Hospitals—a part of Copenhagen University Hospital, The Capital Region, Nordre Fasanvej 57, DK-2000 Frederiksberg, Copenhagen, Denmark; E-Mail: mette.rasmussen.03@regionh.dk; 3 Institute of Preventive Medicine, Bispebjerg and Frederiksberg Hospitals—a part of Copenhagen University Hospital, The Capital Region, Nordre Fasanvej 57, DK-2000 Frederiksberg, Copenhagen, Denmark; E-Mail: berit.lilienthal.heitmann@regionh.dk; 4 National Institute of Public Health, University of Southern Denmark, Øster Farimagsgade 5A, DK-1353 Copenhagen K, Denmark

**Keywords:** smoking cessation, abstinence, heavy smokers, intensive program, nationwide database, group program, individual program, Denmark

## Abstract

*Background*: High-intensity smoking cessation programs generally lead to more continuous abstinence, however, lower rates of success have been reported among heavy smokers. The aim was to evaluate continuous abstinence among heavy smokers during the intensive 6-week Gold Standard Program (GSP) and to identify modifiable factors associated with continuous abstinence. *Methods*: In this nationwide clinical study based on 36,550 smokers attending an intensive cessation program in Denmark. Heavy smoking was defined as ≥7 points in the Fagerström Nicotine Dependency Test, smoking ≥20 cigarettes daily or ≥20 pack-years. *Results*: Overall, 28% had a Fagerström score ≥7 points, 58% smoked ≥20 cigarettes daily and 68% smoked ≥20 pack-years. Continuous abstinence was 33% in responders (6-months response rate: 78%); however, abstinence was approximately 1–6% lower in the heavy smokers than the overall population. Attending GSP with an individual format (*vs.* group/other, OR 1.23–1.44); in a hospital setting (*vs.* pharmacy/municipality services, OR 1.05–1.11); and being compliant (attending the planned meetings OR 4.36–4.89) were associated with abstinence. Abstinence decreased in a dose-dependent manner with increasing smoking severity. *Conclusions*: Abstinence after GSP was 1–6% lower in the heavy smokers than in the overall study population. Modifiable factors may be used for small improvements in continued abstinence. However attempts to improve compliance seemed especially promising.

## 1. Introduction

The health consequences of severe tobacco addiction and an increased rate of tobacco consumption are tremendous [1,2]. There are no generally agreed-upon definitions of heavy smoking, but a cumulative dose of 73,000–146,000 cigarettes, which corresponds to 20 cigarettes per day over 10–20 years, or 10–20 pack-years, is associated with a clinically relevant increase in morbidity [1,2,3]. For clinical purposes, the Fagerström Test for Nicotine Dependence (FTND) [4] and daily cigarette consumption are two measures that are frequently used to guide therapy. The cut-off values of 4 to 7 points (10 points max) and 15, 20 or 25 cigarettes per day often define heavy smokers [5,6,7,8,9,10,11,12].

Heavy smokers are less likely to successfully quit smoking [6,7,12,13,14,15], but pharmacotherapy tailored to the individual level of dependency increases sustained abstinence, especially in patients with a high dependency [9,16]. More intensive and longer-lasting smoking cessation programs results in higher rates of continuous abstinence than less intensive interventions, such as brief advice or motivational interviews [2,16,17,18,19,20,21,22].

The most frequently used intensive intervention program in Denmark is manual-based. The Danish Cancer Society teaches and trains counsellors nationwide for the program. It is offered in a variety of public and private settings, including hospitals, pharmacies and municipalities/counties throughout the Danish regions; the programs differed with respect to format (group or individual) and the modality of payment [6,7,10,23,24,25,26,27].

The setting, locality, format and program payment, as well as gender, age and social status may influence the success rate of continued abstinence. How heavy smokers benefit from intensive programs can be evaluated using the comprehensive data from the Danish Smoking Cessation Database [23].

Therefore, the aims of this study were to evaluate the continuous abstinence among heavy smokers who underwent a standardized intensive smoking cessation program that was integrated with pharmacotherapy and to identify whether modifiable factors related to the program were associated with continuous abstinence. Smoking at least 20 cigarettes daily, scoring at least 7 FTND points and accumulating at least 20 pack-years were the characteristics used to define heavy smokers in this study.

To identify the most relevant definition of heavy smokers according to the outcome after the intensive smoking cessation program, a secondary aim evaluated whether continuous abstinence was associated with smoking severity.

## 2. Experimental Section

### 2.1. Study Design

We used a prospective observational study based on a nationwide registry that was established in 2001 to continuously collect data from smoking cessation programs; the data were delivered by the smoking cessation units and included individual follow-up information on continuous abstinence after 6 months. The data were collected until the summer of 2011 [23,26,27]. This project was approved by the Danish Data Protection Agency (2010-41-5463/2000-54-0013) and registered with the Scientific Ethical Committee (H-C-FSP-2010-049).

### 2.2. Outcomes

The primary outcome was continuous abstinence from the intended quit date until 6 months after that date; the last treatment date was used for the cases without an intended quit date. The information on quit rate was collected by questionnaires (mail or telephone) 6 months after the intended quit date; after 2006, these data were exclusively collected by telephone. From 2001 to 2005, at least one reminder was sent. After 2006, the standard operating procedure included four attempts to reach the patient, at least one of which was in the evening. Continued abstinence until the 6 month time point (±1 month) was documented. The secondary outcome was point prevalence at 6 months of non-smoking considering a period of 14 days in 2006 to 2010 and 7 days in 2001–2005.

### 2.3. Participants

**Figure 1:**
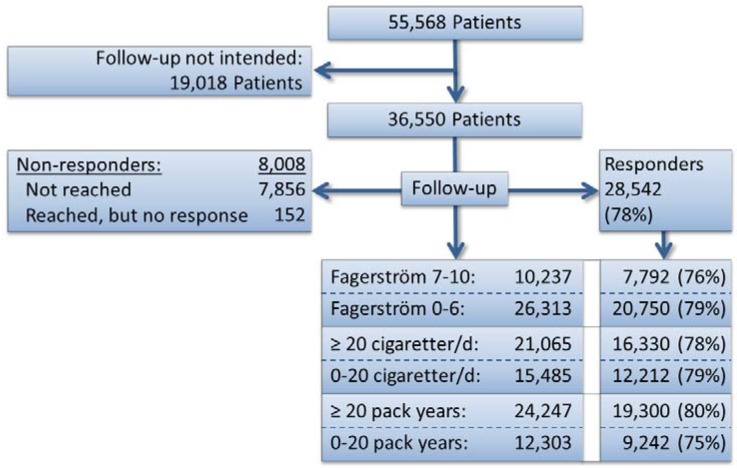
Trial profile.

After providing informed consent, the patients were registered in the Smoking Cessation Database and were included in this study. Among the 67,151 registered interventions from 2001 to 2010, the following were excluded from the study: 1,723 patients because information on their follow-up visits from the last 7 months of the study were not yet available; 76 patients from Greenland; 6,325 patients who did not attend the standardized intensive program; 604 adolescents who were under 18 years of age; and 300 patients who did not provide their age. In addition, the following were also excluded: 1,919 patients who were missing nicotine dependence values; 1,029 patients whose records were missing the number of daily cigarettes; 717 patients who did not list the number of years they had been smoking (resulting in 1,439 patients who were missing information about pack-years); and one patient missing gender information. In total, 55,568 patients had complete information. In addition, some of the smoking cessation units did not follow-up with their patients after 6 months; therefore, patients from these units (*n =* 19,018) were not included in these analyses (Figure 1).

There was no information about the patients’ personal identification number prior to 2006. Therefore, multiple entries could not be sorted. Approximately 4% of the documented interventions were from patients who had attended a program more than once [27].

### 2.4. Setting

During this 10-year study, more than 300 smoking cessation units in Denmark, including those in different settings such as hospitals, primary care and municipality clinics, pharmacies and other private settings, contributed data to the smoking cessation database. In 2007, Denmark was divided into 5 Danish Health Regions corresponding to 14 counties [26].

### 2.5. Intervention

Intensive smoking cessation programs have been standard for smoking intervention in Denmark since 2001. These programs are manual-based teaching sessions (6-week sessions with 5–6 meetings in a group or individual format) with individual pharmocotherapy according to the level of dependence (e.g., individually tailored nicotine replacement therapy according to nicotine dependence measured by Fagerström Score [4], vareniclin or bupropion). These programs were originally developed under the direction of the National Cancer Society together with the previous “National Stop Smoking Centre” [6,7,10,26,27]. The program included a curriculum with topics such as readiness to change and setting a quit date, ambivalence and motivation, withdrawal symptoms and nicotine replacement therapy, relapse prevention and living completely smoke-free. The programs were usually offered free of charge; most of the patients paid for their medication, but a few received free medication. Furthermore, a hotline was available during daytime hours on working days.

### 2.6. Data

Data were collected on an individual’s socio-demographic parameters, smoking history, their intervention program and follow-up results (Table 1).

**Table 1:** Characteristics of the study population allover and by heavy smoking definition^ a^, with continuous abstinence rates for the group of patients responding to follow up (base) and under two scenarios: (1) assuming non-responders relapsed (worst case), and (2) assuming non-responders abstained (best case).

	All			Fagerström score ≥ 7			Cigarettes/day ≥ 20			Pack years ≥ 20			
	*n =*	base	(worst-best)	*n =*	base	(worst-best)	*n =*	base	(worst-best)	*n =*	base	(worst-best)	
**Total**	**36,550**	33%	(26%–48%)	**10,237**	27%	(21%–45%)	**21,065**	30%	(23%–46%)	**24,247**	32%	(25%–46%)	
**Capital Region**	**12,100**	32%	(25%–48%)	**3,504**	26%	(20%–45%)	**7,293**	30%	(23%–46%)	**8,010**	31%	(24%–46%)	
**Central Denmark**	**8,216**	33%	(25%–48%)	**2,183**	27%	(20%–45%)	**4,419**	29%	(22%–45%)	**5,200**	32%	(25%–46%)	
**North Denmark**	**1,477**	36%	(29%–50%)	**368**	30%	(24%–46%)	**780**	33%	(26%–48%)	**982**	36%	(29%–48%)	
**Region Zealand**	**5,144**	34%	(28%–47%)	**1,512**	29%	(23%–43%)	**3,149**	32%	(26%–44%)	**3,687**	33%	(28%–44%)	
**South Denmark**	**9,613**	34%	(27%–48%)	**2,670**	27%	(21%–45%)	**5,424**	31%	(24%–47%)	**6,368**	32%	(26%–46%)	
**Unknown**	**0**												
**Pharmacy**	**9,664**	33%	(26%–48%)	**2,749**	27%	(20%–44%)	**5,592**	29%	(23%–45%)	**6,789**	31%	(25%–45%)	
**Hospital**	**7,813**	36%	(28%–50%)	**2,475**	30%	(22%–47%)	**4,610**	33%	(25%–49%)	**5,040**	35%	(27%–49%)	
**All other settings**	**19,073**	32%	(25%–47%)	**5,013**	27%	(20%–44%)	**10,863**	30%	(23%–45%)	**12,418**	31%	(25%–45%)	
**Individual format**	**4,526**	37%	(27%–53%)	**1,439**	34%	(24%–53%)	**2,612**	34%	(25%–52%)	**2,931**	35%	(27%–51%)	
**Group format**	**31,918**	33%	(26%–47%)	**8,773**	26%	(20%–43%)	**18,401**	30%	(23%–45%)	**21,260**	32%	(25%–45%)	
**All other formats**	**106**			**25**			**52**			**56**			
**No free medication/other**	**15,112**	33%	(26%–47%)	**3,894**	27%	(21%–43%)	**8,400**	30%	(24%–44%)	**10,008**	32%	(26%–44%)	
**Free medication for days**	**16,478**	32%	(25%–48%)	**4,766**	26%	(20%–45%)	**9,722**	29%	(22%–46%)	**10,904**	31%	(25%–46%)	
**Free for <5 weeks**	**4,026**	36%	(27%–50%)	**1,293**	31%	(24%–48%)	**2,416**	34%	(26%–50%)	**2,775**	35%	(28%–49%)	
**Free for the total course**	**934**	40%	(32%–53%)	**284**	35%	(27%–50%)	**527**	37%	(29%–51%)	**560**	36%	(30%–48%)	
**Women**	**22,538**	32%	(25%–46%)	**5,620**	25%	(19%–42%)	**11,423**	27%	(21%–43%)	**13,851**	29%	(24%–43%)	
**Men**	**14,012**	36%	(28%–50%)	**4,617**	30%	(23%–48%)	**9,642**	34%	(26%–49%)	**10,396**	36%	(28%–49%)	
**Unknown**	**0**												
**18-24 years age**	**1,407**	26%	(17%–54%)	**280**	19%	(11%–53%)	**626**	22%	(14%–52%)	**7**	–	–	–
**25-34 years**	**4,855**	32%	(23%–50%)	**1,164**	23%	(16%–46%)	**2,341**	28%	(20%–49%)	**683**	26%	(19%–46%)	
**35-44 years**	**8,038**	33%	(25%–48%)	**2,446**	26%	(19%–45%)	**4,710**	29%	(22%–46%)	**4,784**	29%	(22%–46%)	
**45-54 years**	**10,135**	33%	(26%–47%)	**3,277**	27%	(21%–44%)	**6,316**	30%	(23%–45%)	**8,207**	31%	(25%–46%)	
**55-64 years**	**8,946**	35%	(29%–47%)	**2,524**	30%	(24%–44%)	**5,492**	33%	(27%–45%)	**7,851**	34%	(28%–46%)	
**65+ years**	**3,169**	34%	(28%–46%)	**546**	32%	(26%–45%)	**1,580**	33%	(27%–45%)	**2,715**	33%	(27%–45%)	
**Unknown**	**0**												
**No previous attempt**	**13,168**	32%	(25%–47%)	**4,323**	27%	(20%–45%)	**8,351**	30%	(23%–46%)	**9,371**	32%	(25%–46%)	
**Previous attempt**	**22,741**	34%	(27%–48%)	**5,703**	27%	(21%–44%)	**12,332**	30%	(24%–46%)	**14,395**	32%	(26%–46%)	
**Unknown**	**641**			**211**			**382**			**481**			
**Living with smoker**	**13,291**	32%	(25%–46%)	**3,912**	27%	(21%–43%)	**8,059**	30%	(23%–45%)	**8,762**	31%	(25%–51%)	
**Not living with smoker**	**22,975**	34%	(26%–49%)	**6,233**	28%	(21%–45%)	**12,825**	31%	(23%–47%)	**15,293**	32%	(25%–47%)	
**Unknown**	**284**			**92**			**181**			**192**			
**Compliant**	**23,400**	42%	(34%–52%)	**6,144**	37%	(30%–49%)	**12,990**	40%	(32%–51%)	**15,919**	40%	(34%–50%)	
**Not compliant**	**12,677**	15%	(11%–40%)	**3,968**	10%	(7%–39%)	**7,805**	12%	(9%–38%)	**8,032**	13%	(9%–37%)	
**Unknown**	**473**			**125**			**270**			**296**			
**Employed**	**24,677**	34%	(27%–49%	**6,694**	29%	(22%–45%)	**14,040**	31%	(24%–47%)	**15,477**	33%	(26%–47%)	
**Not employed**	**10,941**	31%	(24%–46%)	**3,285**	24%	(18%–44%)	**7,025**	28%	(22%–44%)	**8,770**	30%	(24%–45%)	
**Unknown**	**698**			**195**			**395**			**465**			
**2006-2010**	**21,516**	33%	(26%–46%)	**5,577**	27%	(21%–43%)	**12,172**	29%	(23%–44%)	**14,011**	31%	(25%–44%)	
**2001-2005**	**15,034**	34%	(26%–50%)	**4,660**	28%	(21%–47%)	**8,893**	31%	(24%–48%)	**10,236**	33%	(26%–48%)	
**Unknown**	**0**												

^a^ Heavy smokers: Fagerström Test for Nicotine dependence score of at least 7, or smoking at least 20 cigarettes per day (20 CPD) or at least 20 pack years.

Program compliance was defined as having attended at least 75% of the scheduled meetings, as documented by the units. To account for possible administrative effects and historical, social and technical changes in the setting, which included a reform of the healthcare system in 2007, the study period was categorized as “2001–2005” and “2006–2010”.

### 2.7. Statistical Methods

Continuous abstinence was used based on the patients’ self-reports. To compare the results from this observational prospective study with results from randomized controlled studies, the abstinence was reported according to the Russell criteria [28], assuming in the worst case that all non-responders had relapsed. The best case results assumed that none of the non-responders had relapsed. These assumptions were used to estimate the range of confidence.

Multiple logistic regression analyses were used to test for differences in quit rates by first entering all of the predictors together, followed by a stepwise backward procedure with the criterion P(in) < 0.10. The results were presented as odds ratios (OR) and the corresponding 95% confidence intervals (CI). The results were considered significant if the CI did not include the value 1. For the logistic regression, dummy variables as independent variables were used, as outlined in Table 1.

**Figure 2:**
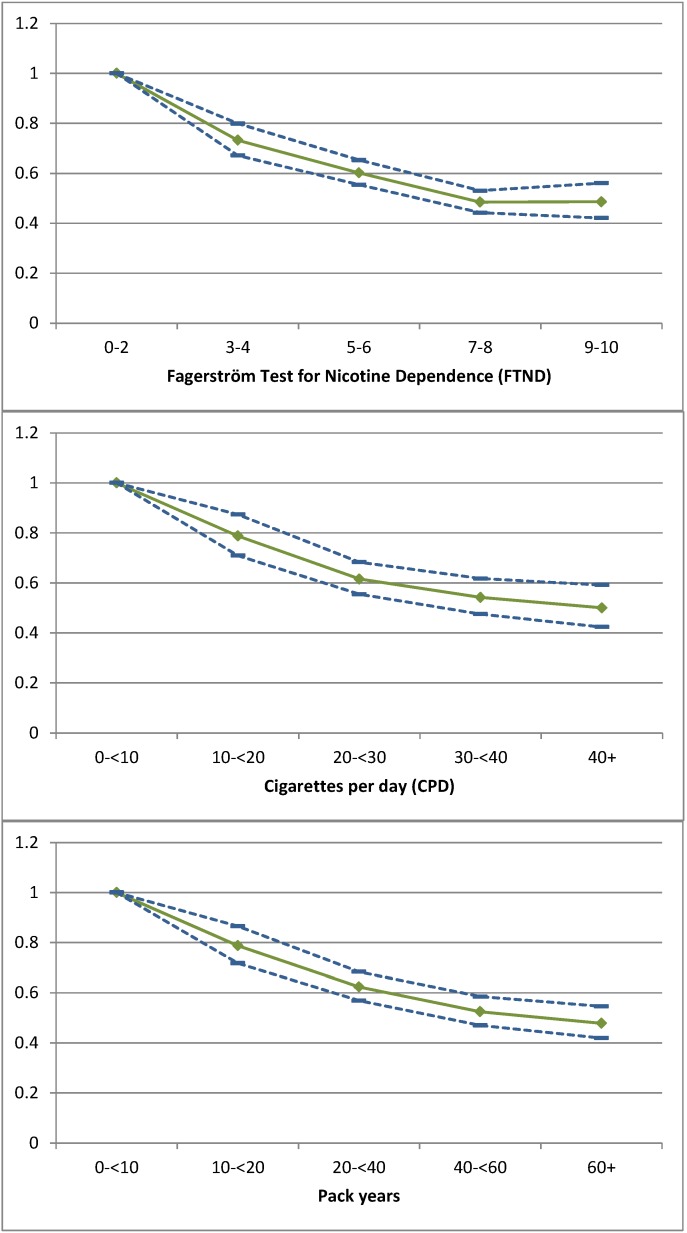
Odds ratio (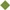) and 95% CI (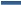) of continuous abstinence as the dependent variable for Fagerström points (FTND), cigarettes per day (CPD) and pack-year after adjusting (final model) for setting, region, format, payment modality, age, gender, employment, attempts to quit, living with a smoker, compliance and calendar period.

To evaluate the relationship between the quit rate and smoking severity using secondary analysis, patients were categorized by their smoking severity according to the FTND [4,14], the number of daily cigarettes smoked and the number of pack-years as shown in Figure 2.

In addition, the entry characteristics of the patients included in the primary outcome analyses were compared with those of the patients not included in the analyses. All statistical calculations were performed using PASW 19 (IBM Corporation, Somers, NY, USA).

## 3. Results

Documented follow-up data were available for 28,542 interventions for the responding patients (Figure 1); among these, 28% reported 7 or more points on the FTND scale, 58% had smoked at least 20 cigarettes per day and 68% smoked at least 20 pack-years, thereby fulfilling the criteria for heavy smoking.

The patient characteristics are shown in Table 1. The analyses for the 6-month follow-up visit included both the responders and the non-responders; out of all the responding patients, 9,490 (33%) reported continuous abstinence. The “worst case” quit rate, when all of the non-responders were considered smokers, was 26%. However, the “best case” quit rate, when all of the non-responders were considered non-smokers, was 48%.

The heavy smokers had a significantly lower rate of continuous abstinence compared with the 33% abstinence in all of the patients; there was a 6% (2–9% in the subgroups) difference in the Fagerström Tests of the patients with at least 7 points; 3% (1–5%) difference in the smokers that smoked at least 20 cigarettes per day; and 1% (0–4%) for that smoked at least 20 pack-years (Table 1). The results of the multivariate analysis for the heavy smokers are shown in Table 2.

**Table 2:** Multivariate model and final model after backward elimination for continuous abstinence as the dependent variable. The independent categorical variables were given with the reference category in brackets. (Significant results were additionally marked with an * asterisk.)

	Fagerström score ≥ 7	Cigarettes/day ≥ 20	Pack years ≥ 20
	ORs (95% CI)	Exp(B) (95% CI)	Exp(B) (95% CI)
**MULTIVARIATE**			
Capital (*vs.* other regions)	0.99 (0.87–1.13)	1.00 (0.92–1.08)	0.94 (0.87–1.01)
(Setting)		*	*
Pharmacy (*vs.* other settings)	0.89 (0.78–1.02)	0.91 (0.83–0.99) *	0.90 (0.83–0.98) *
Hospital (*vs.* other settings)	1.05 (0.91–1.22)	1.11 (1.01–1.23) *	1.10 (1.00–1.20) *
Pharmacy (*vs.* hospitals)	0.85 (0.72–1.00)	0.82 (0.73–0.91) *	0.82 (0.74–0.91) *
Individual (*vs.* other formats)	1.44 (1.23–1.68) *	1.29 (1.15–1.44) *	1.22 (1.10–1.35) *
Payment for weeks (*vs.* non/shorter payments)	1.06 (0.78–1.43)	1.08 (0.87–1.34)	0.98 (0.80–1.20)
Men (*vs.* women)	1.25 (1.12–1.39) *	1.31 (1.22–1.41) *	1.29 (1.21–1.38) *
Age (every 10 years)	1.07 (1.02–1.13) *	1.05 (1.02–1.09) *	1.10 (1.06–1.14) *
Compliant (*vs.* other)	4.90 (4.28–5.61) *	4.38 (4.01–4.78) *	4.35 (4.01–4.73) *
Living with a smoker (*vs.* not)	0.94 (0.84–1.05)	0.95 (0.89–1.03)	0.95 (0.89–1.01)
No earlier attempts (*vs.* earlier attempts)	1.03 (0.93–1.15)	0.99 (0.92–1.07)	1.03 (0.97–1.10)
Employed (*vs.* other)	1.28 (1.13–1.45) *	1.22 (1.12–1.33) *	1.25 (1.16–1.36) *
2001–2005 (*vs.* 2006–2010)	0.85 (0.76–0.96) *	0.87 (0.80–0.94) *	0.88 (0.82–0.95) *
Constant	0.08 *	0.10 *	0.09 *
**FINAL MODEL**			
(Setting )		*	*
Pharmacy (*vs.* other settings)	0.90 (0.78–1.02)	0.91 (0.83–0.99) *	0.92 (0.85–0.99) *
Hospital (*vs.* other settings)	1.05 (0.91–1.21)	1.11 (1.01–1.23) *	1.07 (0.98–1.17)
Pharmacy (*vs.* hospitals)	0.85 (0.73–0.99)	0.81 (0.73–0.90) *	0.86 (0.78–0.94) *
Individual (*vs.* other formats)	1.44 (1.23–1.68) *	1.30 (1.16–1.45) *	1.23 (1.11–1.36) *
Men (*vs.* Women)	1.25 (1.12–1.39) *	1.31 (1.22–1.41) *	1.29 (1.21–1.38) *
Age (every 10 years)	1.07 (1.02–1.13) *	1.05 (1.02–1.09) *	1.10 (1.06–1.14) *
Compliant (*vs.* other)	4.89 (4.27–5.60) *	4.38 (4.01–4.78) *	4.36 (4.01–4.73) *
Employed (*vs.* Other)	1.28 (1.13–1.44) *	1.22 (1.12–1.33) *	1.25 (1.16–1.35) *
2001–2005 (*vs.* 2006–2010)	0.85 (0.76–0.95) *	0.87 (0.80–0.94) *	0.89 (0.83–0.95) *
Constant	0.07 *	0.10 *	0.09 *

The final models suggest that an “individual format” was the only modifiable factor that was consistently associated with an increase in continuous abstinence after 6 months, regardless of which “heavy smoking” definition was used. The rate for continuous abstinence was higher among the patients who attended cessation programs in a hospital setting than among the patients who attended programs in pharmacies or community settings. However, results were not similar for the heavy smokers with at least 7 FTND points. Other indicators of continuous abstinence were male gender, increased age, high compliance, being employed and calendar period.

### 3.1. Quit Rate and Severity of Smoking

The rates for continuous abstinence differed substantially in a dose-response manner by level of smoking based on FTND score, ranging from 43% (34%–55%) in the group with 0–2 points to 27% (20%–46%) in the group with 9–10 points. Those in the different groups based on the number of cigarettes per day and pack-years had similar dose responses. The dose response was present for the unadjusted raw quit rates and for the odds ratios in the model that adjusted for age, gender, setting, format, region, payment modalities, number of previous attempts to quit, living with a smoker, compliance and calendar period (Figure 2). The overall continuous abstinence rate was 33%. Compared with this 33%, all of the patients with at least 5 FTND points, who smoked at least 20 cigarettes per day or smoked at least 20 pack-years, had lower continuous abstinence rates.

### 3.2. Point Prevalence

Data on the point abstinence prevalence were available for 28,574 documented interventions. Overall, this point abstinence prevalence was approximately 4–5% higher than the continuous quit rate: 11,106 (overall, 39%; worst case, 30%; best case, 52%) interventions resulted in patient-reported point abstinence; the patients from 2001 to 2005 had a 7-day point abstinence prevalence of 38%, whereas the patients from 2006 to 2010 had a 14-day point abstinence prevalence of 39%.

Overall, the point abstinence prevalence was significantly lower in the heavy smokers: 32% (25%–48%) in patients with 7–10 FTND points, 35% (28%–50%) in the group that smoked 20 or more cigarettes per day and 37% (30%–50%) in those reporting 20 or more pack-years.

### 3.3. Sensitivity Analyses

The proportion of non-responders was 22% (Figure 1). The sensitivity analyses showed that non-responders were more likely to be patients who attended individual programs (25%), young patients (18–24 years: 37%, 25–34 years: 27%), patients with low pack-years (0–10 years: 28%) and non-compliant patients (29%). By contrast, the proportion of non-responders was lower among the patients from Region Zealand (19%) and among the elderly patients (55–64 years: 19%, 65 and more: 18%).

Overall, 34% of the non-responders originated from units that did not perform patient follow-ups. Follow-up increased during the course of the study: only 58% of the patients who were registered prior to 2006 participated in the follow-ups, but 72% participated after 2006. Patients who originated from the units that did not have follow-up were mainly from regions outside of the Capital Region (39%* vs.* 22% Capital region, *p <* 0.001), and they were more likely to attend individual programs (41%* vs.* 33%, *p <* 0.001) and were less likely to receive free medication (35%* vs.* 31%, *p <* 0.001).

## 4. Discussion

The present study had two main findings. First, this study showed that after attending intensive smoking cessation programs, heavy smokers had a 1–6% lower continuous abstinence rate than the 33% overall quit rate.

Continuous abstinence consistently decreased in a dose-dependent manner with increased smoking severity; the decrease in continuous abstinence was measured by the amount of cigarettes smoked per day, pack-years and nicotine dependency. These results highlight that no clear cut-offs exist for heavy smokers. Using 33% as the percent of patients who demonstrated continued abstinence, the cut-off value for the FTND score would be 5 instead of the 7 points used for the definition of heavy smoking in this paper. The other definitions used for heavy smokers would remain the same. The new cut-off values add to the many other definitions of heavy smoking [2,5,6,7,8,9,10,11,12] but are closely related to the intensity of the program, and other interventions may result in different cut-off points.

In this study, compliance was defined as attending five education meetings and was the overall strongest predictor of a favourable outcome. In the literature, continuous abstinence is consistently associated with increased intervention duration [2,15,17,29,30]; however, our findings together with findings from randomized controlled trials underline the importance of completing the cessation program [16,17,18,19,20,21,22,31]. Other modifiable factors that were good predictors included an individual and hospital setting/format.

Previously, group format and one-on-one settings were equally effective in achieving abstinence [18,19,31,32,33]. However, other reports suggested that the group format is superior [15,29]. In Denmark, nine out of 10 interventions are delivered in a group format, whereas in the UK, the group format is only used by a small percentage of treatment-seeking smokers [15,29,34]. Obstacles to delivering smoking cessation intervention in groups include patient preferences and organizational and feasibility issues [15,29,34]. However, these obstacles do not seem to be of equal importance in Denmark.

In addition, non-modifiable factors, such as older age, male gender and not living with a smoker, were all associated with a higher rate of continuous abstinence after 6 months, which has been reported in previous studies [1,2,6,7,10,12,15,29,35,36,37].

To the best of our knowledge, this is the first study that addresses the impact of region, setting, format and payment modality on continuous abstinence in heavy smokers.

In their daily practice, clinicians should ensure that smokers receive treatment to address not only smoking in general but also individual background. Clinicians should emphasize that completing an intensive program is associated with successful quitting for 1 out of 3 or 4 smokers, even the heavy smokers.

This study had several limitations. Relevant selection, information availability, attrition bias and confounding must be considered. The allocation of a patient into a single patient format, setting, region or payment modality may have been influenced by factors not sufficiently addressed, as this was not a randomized controlled trial. In addition the outcomes were influenced by the study period which was included in the analysis. In the later part of the study, more units conducted patient follow-ups, and guidelines changed from written follow up to follow up by phone. In addition, because all of the patients were treatment seekers and were registered in an intensive program within the Danish health system, the findings of this study may be limited in their generalizability to other heavy smokers. The continuous abstinence rates reported may be overestimated by approximately 3 to 6% [15,36,38,39] because this outcome relied on self-reports. The proportion of patients who did not respond to follow-ups was moderate, and the sensitivity analyses revealed a relatively robust finding. Few patients received free medications for more than a week, so it was not possible to draw conclusions about the potential benefits of providing free medications. Other factors that may have influenced the outcome but were not part of the study included co-morbidity, control of stress, tension, depressive or anxious moods, social support, motivation, and using smoking to control the body weight [5,35,37,40,41,42,43,44]. The proportion of patients attending a program more than once (approximately 4%) was considered small.

Strength of this evaluation of smokers in intensive programs was that it included approximately 90% of all smoking cessation intervention activities in Denmark. More than 1% of the total Danish population or more than 5% of all Danish smokers have been registered in this smoking cessation database to date, regardless of smoking severity, motivation to quit or co-morbidity or whether a quit-date was set. These characteristics are often used as selection criteria in randomized control trials [15,45]. Therefore, registry-based studies are needed to study smoking cessation interventions.

Higher continuous abstinence rates are higher in more intensive programs [17,30] than in programs that are shorter in duration. In general, the reported differences in the continuous abstinence rates after smoking cessation intervention with respect to smoking severity vary greatly, from smaller clinically insignificant differences to differences exceeding a factor of 3 [4,9,14,15,46,47,48]. This difference may be due to the intensity of the programs or due to cultural differences, patient preferences, motivation, staff competencies and other factors. Finally, this study emphasizes that heavy smokers benefit from more intensive programs with individualized approaches than other programs.

## 5. Conclusions

Nationwide data delivered to the Danish Smoking Cessation Database by smoking cessation units which included individual follow-up information on continuous abstinence after 6 months demonstrated that abstinence after the intensive 6-week Gold Standard Program was 1–6% lower in the heavy smokers than in the overall study population. More heavy smokers report continuous abstinence after attending the planned meetings of intensive programs with individualized approaches compared to programs in a group format or compared to patients with a lower compliance. For some heavy smoking patients abstinence was higher in hospital programs and lower in pharmacy based programs. The likelihood of abstinence decreased also in a dose-dependent manner with increasing smoking severity.
